# SPK1-transfected UCMSC has better therapeutic activity than UCMSC in the treatment of experimental autoimmune encephalomyelitis model of Multiple sclerosis

**DOI:** 10.1038/s41598-018-19703-5

**Published:** 2018-01-29

**Authors:** Yun-Liang Wang, Peng Xue, Chun-Yang Xu, Zhen Wang, Xin-Shan Liu, Lin-Lin Hua, Hong-Ying Bai, Zhi-Lei Zeng, Hai-Feng Duan, Jin-Feng Li

**Affiliations:** 1 Department of Neurology, the Second Affiliated Hospital of Zhengzhou University, No. 32 Nanyang Road, Zhengzhou, 450014 China; 2 Department of Neurology, the 148th Hospital of Chinese PLA, No. 20 North Road Zhoucun District, Zibo, 255300 China; 3 Electroencephalogram Room of Sanbo Brain Hospital, Capital Medical University, No. 50 Xiangshanyikesong Haidian District, Beijing, 100093 China; 4 Department of Experimental Hematology, Beijing Institute of Radiation Medicine, Beijing, 100850 China; 5 Department of Medical Oncology, Chinese PLA General Hospital, Beijing, 100853 China

## Abstract

Multiple Sclerosis (MS), is a chronic inflammatory autoimmune disorder of the central nervous system that leads to chronic demyelination with axonal damage and neuronal loss. Mesenchymal stem cells (MSCs) represent a promising therapeutic approach for MS. In the current study, we investigated the effects of MSCs derived from the human umbilical cord (UCMSC) transfected by sphingosine kinase 1 (SPK1) gene. All the results showed that transplantation of UCMSCs gene modified by SPK1 (UCMSC-SPK1) dramatically reduce the severity of neurological deficits of the experimental autoimmune encephalomyelitis (EAE) mice, paralleling by reductions in demyelination, axonal loss, and astrogliosis. UCMSC-SPK1 transplantation also could inhibit the development of natural killer (NK) responses in the spleen of EAE mice, and increase the ratio of CD4^+^ CD25^+^ FoxP3^+^ (Treg) T cells. Furthermore, we described that a shift in the cytokine response from Th1/Th17 to Th2 was an underlying mechanism that suppressed CNS autoimmunity. UCMSCs transfected by SPK1 gene potentially offer a novel mode for the treatment of MS, and the specific mechanism of SPK1 in treating MS/EAE.

## Introduction

Multiple sclerosis (MS), a chronic inflammatory demyelinating disease of central nervous system (CNS), generally manifests in an initial relapsing-remitting clinical course that culminates in permanent neurological damage^1,2^. The main pathological features of the disease include focal CNS inflammation with axonal demyelination and neuronal death^1,3^. The common clinical strategy for therapy of acute relapses in MS is either by high dose, short-term pulse therapy with glucocorticoid^4^ or by immunomodulatory treatments such as interferon beta (IFN-β)^5^, glatiramer acetate^6^, and mitoxantrone^7^. Although these drugs can slow down MS progression and ameliorate intensity of relapsed disease, however, long-term therapy with these drugs often gives rise to significant adverse effects including depression, infection, cardiotoxicity, nausea and anemia^4–8^. Therefore, new therapy with high efficacy but low side-effect is urgently needed for MS treatment.

Emerging data suggested that stem cells have great potential in regenerative medicine, and such cell-based therapy may provide an alternative approach to the currently approved MS treatments^9–11^. Mesenchymal stem cells (MSCs) are adult stem cells typically found in the bone marrow, and in other tissues including umbilical cord (UC), fetal liver, and adipose tissue. MSCs are considered to be multipotent, and have anti-inflammatory as well as regenerative properties^9–11^. MSCs derived from the human umbilical cord (UCMSC) are more primitive, and possess multiple advantages including ethical agreeableness, a less invasive procedure for isolation, low immunogenicity, high proliferation capacity, and multi-lineage differentiation capability^12–14^. Several studies have used MSC to modulate immune responses in autoimmune diseases including rheumatoid arthritis, type 1 diabetes, and encephalomyelitis^15–17^. UCMSCs are immune naïve and they are able to differentiate into other phenotypes, including the neural lineage. In the report of Rahyussalim AJ, UCMSC implantations led to significant improvement for spinal cord entrapment and kidney failure^18^. Therefore, it is reasonable to hypothesize that UCMSC may have a significant therapeutic effect on MS.

Sphingosine 1-phosphate (S1P), the product of sphingosine phosphorylation, is mainly catalyzed by one of the sphingosine kinase isoenzymes, sphingosine kinase 1 (SPK1)^19^. Numerous studies have shown that S1P signaling orchestrates many important physiological and pathophysiological processes including cell proliferation, migration and immune regulations^19^. S1P works as a ligand for a subset of G protein coupled receptor (S1PR) proteins, and functions on various cellular events including neurogenesis, angiogenesis, and immune response^20^. S1PR modulator FTY720 (Fingolimod/Gilenya) is a sphingosine-related molecule exhibiting an immunomodulatory function, which has recently been approved as an oral treatment for relapsing forms of MS^20,21^. Therefore, we speculate that SPK1 may enhance therapeutic efficiency of UCMSC therapy for experimental autoimmune encephalomyelitis (EAE). The purpose of this study was to observe the effect of UCMSC transfected by SPK1 gene on experimental autoimmune encephalomyelitis (EAE), a common animal model to study the pathogenesis and therapeutic interventions of MS, due to the recombinant adenovirus carrying SPK1 gene having already been constructed in our lab.

Therefore, in the current study, we tried to use UCMSC as SPK1 gene delivery vehicles with CNS targeting migration capabilities and evaluated the therapeutic efficiency of SPK1-secreting UCMSC (UCMSC-SPK1) in EAE. The results demonstrated that UCMSC transfected by SPK1 can be genetically engineered to secrete concentrations of SPK1. Furthermore, UCMSC-SPK1 transplanted during the priming phase of EAE were able to reduce the inflammatory immune response, skew the proinflammatory cytokine profile and suppress disease severity. These data indicate that UCMSCs gene modified by SPK1 may provide another tool in the armamentarium of cellular and gene therapy approaches being developed for the treatment of MS.

## Results

### UCMSC highly expressed SPK1 after transduction with Ad-SPK1

**Figure 1:**
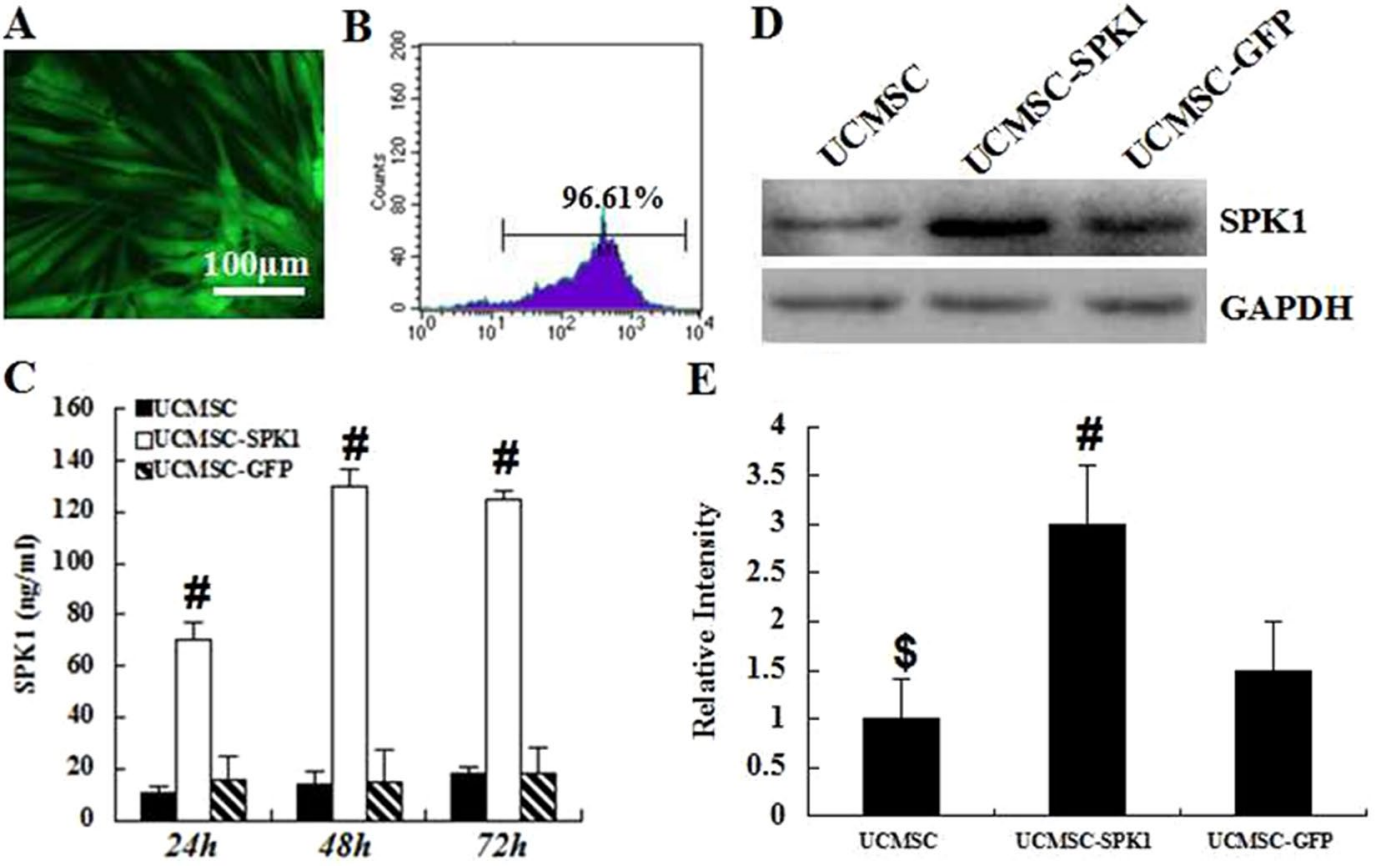
The expression of SPK1 in transduced UCMSC *in vitro*. (**A**) Expression of GFP in UCMSC transfected by Ad-GFP as determined by fluorescence microscopy. (**B**) Flow cytometric analysis of GFP expression in UCMSC transfected by Ad-GFP. (**C**) Production of bioactive SPK1 in the supernatant of non-transduced (UCMSC) and transduced (UCMSC-SPK1). Supernatant from cultured cells was retrieved on the indicated time points, processed and analyzed using a mousespecific SPK1 ELISA assay. (**D**) Typical bands of SPK1 and GAPDH detected by western blotting. (**E**) Gray intensity analysis of SPK1. All data are presented as mean ± standard error of mean. (^*#*^*P* < 0.01, UCMSC-SPK1 vs UCMSC and UCMSC-GFP, ^$^*P* < 0.01 UCMSC vs UCMSC-SPK1).

Firstly, the UCMSC were transduced by Ad-GFP, the fluorescence microscopy and flow cytometry showed that greater than 90% of cells expressed GFP (Fig. 1A,B). Ad-SPK1 are efficiently express high levels of transgenic SPK1 in UCMSC. As shown in Fig. 1C, SPK1 accumulated to about 70 ng/ml in the supernatant at 24 h in Ad-SPK1 groups. It gradually increased, peaking at 130 ng/ml at 48 d. At 72 d, concentration of SPK1 declined slightly to approximately 125 ng/ml. In blank control and Ad-GFP groups, SPK1 concentration remained stable at about 15 ng/ml. It is clear that SPK1 protein level obviously increased after transduction with Ad-SPK1 than UCMSC alone and transduction with Ad-GFP at different time-point (^*#*^*P* < 0.01). Furthermore, the expression of SPK1 was detected by a Western blot assay, and the results was consistent with that of the ELISA (Fig. 1D,E).

### Neurological function evaluation

**Figure 2:**
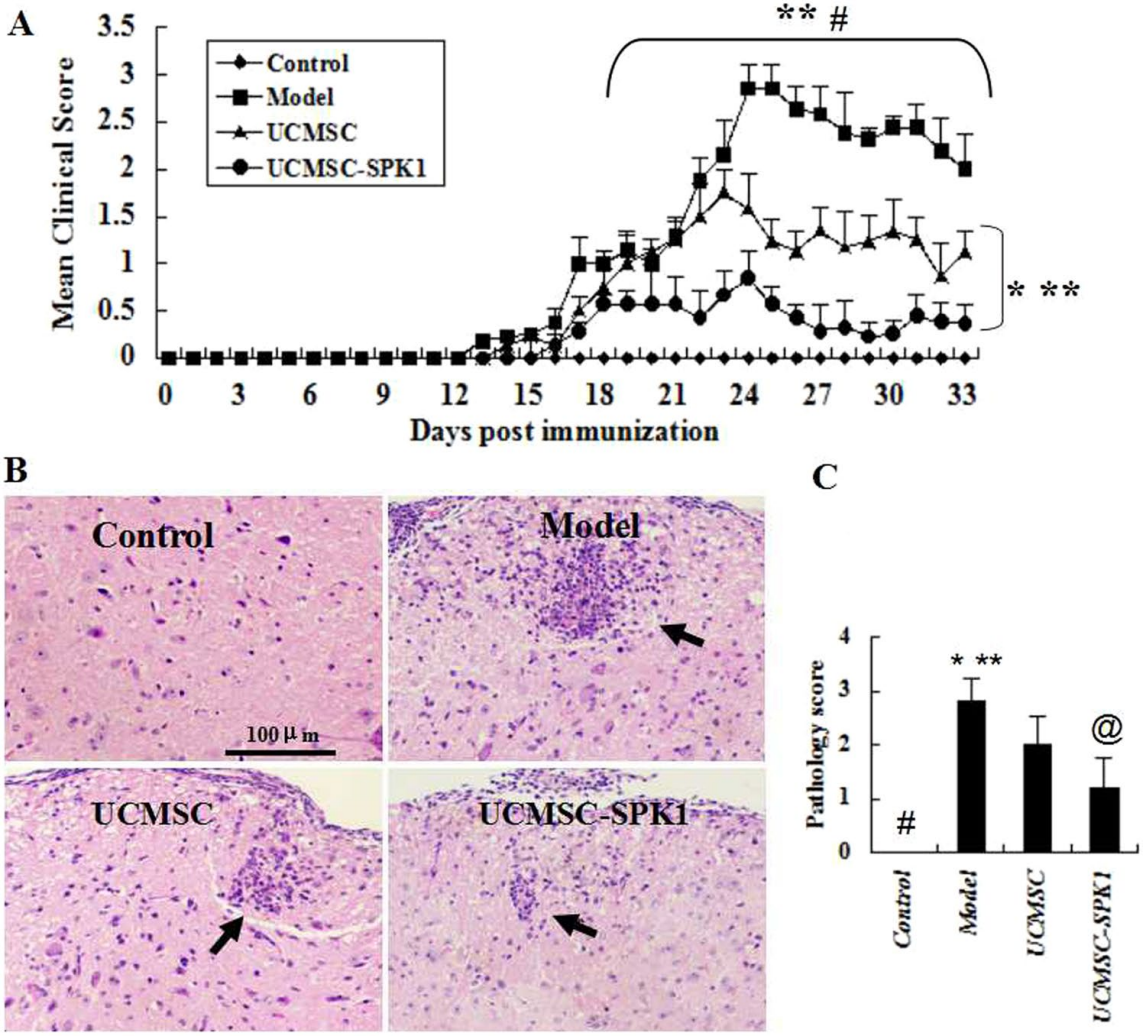
The mean clinical score and histopathological analysis. (**A**) Daily mean of the clinical scores shows a statistical difference between the UCMSC-SPK1 group and model group mice starting from 17 dpi (**P < 0.01). While from 25 dpi, there were significantly difference between the UCMSC-SPK1 group and UCMSC group (^*#*^*P* < 0.01). From 17 dpi, the mean clinical scores of UCMSC-SPK1 group and UCMSC group always were higher than that of the control group (*P < 0.05, **P < 0.01). (**B**) In spinal cord sections, mononuclear cell infiltration was observed in the injured area of EAE mice (↑), obviously decreased in UCMSC-SPK1 mice group as compared to UCMSC mice group. (**C**) The pathology score (^*#*^*P* < 0.01 the control vs the model group, *P < 0.05 the model group vs UCMSC group, **P < 0.01 the model group vs UCMSC-SPK1 group, ^*@*^*P* < 0.05 UCMSC group vs UCMSC-SPK1 group).

To examine the effects of UCMSC-SPK1 administration on the clinical course of EAE, we used a chronic progressive disease model induced by immunizing female C57Bl/6 mice with MOG_35–55_. From 13 d.p.i., some mice started to show signs of EAE onset, which were characterized by the appearance of a flaccid tail and paralysis of the hind legs. These symptoms gradually aggravated between days 16 and 27, leading to complete paralysis, tetraplegia, and even moribundity. Our results showed that the mean daily clinical score was reduced in mice receiving UCMSC-SPK1 and UCMSC compared with that of the model mice from 19 and 24 d.p.i, respectively (**, ^*#*^*P* < 0.01). There were significant differences of mean daily clinical score between UCMSC-SPK1 group and UCMSC group from 20 d.p.i (*P < 0.05, **P < 0.01, Fig. 2A). Interestingly, treatment with UCMSC or UCMSC-SPK1 also could reduce or prevent the disease-related mortality of EAE mice (Table 1).

**Table 1:** Clinical features of EAE mice.

Treatment	Disease incidence	Mortality	Disease onset (d)	Maximum score	Mean score
Control	12/12	5/12	12.8 ± 0.1	2.85 ± 0.2	1.83 ± 0.8
UCMSC	12/12	3/12	13.9 ± 0.4	1.75 ± 0.3	1.07 ± 0.6
UCMSC-SPK1	12/12	1/12	15.8 ± 0.7	0.8 ± 0.7	0.48 ± 0.2

### Pathological changes on spinal cord tissues

To compare the lesion formation in the spinal cords of UCMSC-SPK1 mice group to that of UCMSC mice group, we used HE staining for observing cell infiltration and LFB staining for observing demyelination. In spinal cord sections, we found that mononuclear cell infiltration into the injured area during MOG_35–55_-induced EAE (↑), obviously decreased in UCMSC-SPK1 group as compared to UCMSC group (Fig. 2B). The pathology score was shown in Fig. 2C (^*#*^*P* < 0.01 the control vs the model group, *P < 0.05 the model group vs UCMSC group, **P < 0.01 the model group vs UCMSC-SPK1 group, ^@^P < 0.05 UCMSC group vs UCMSC-SPK1 group).

### The observation of demyelination

**Figure 3:**
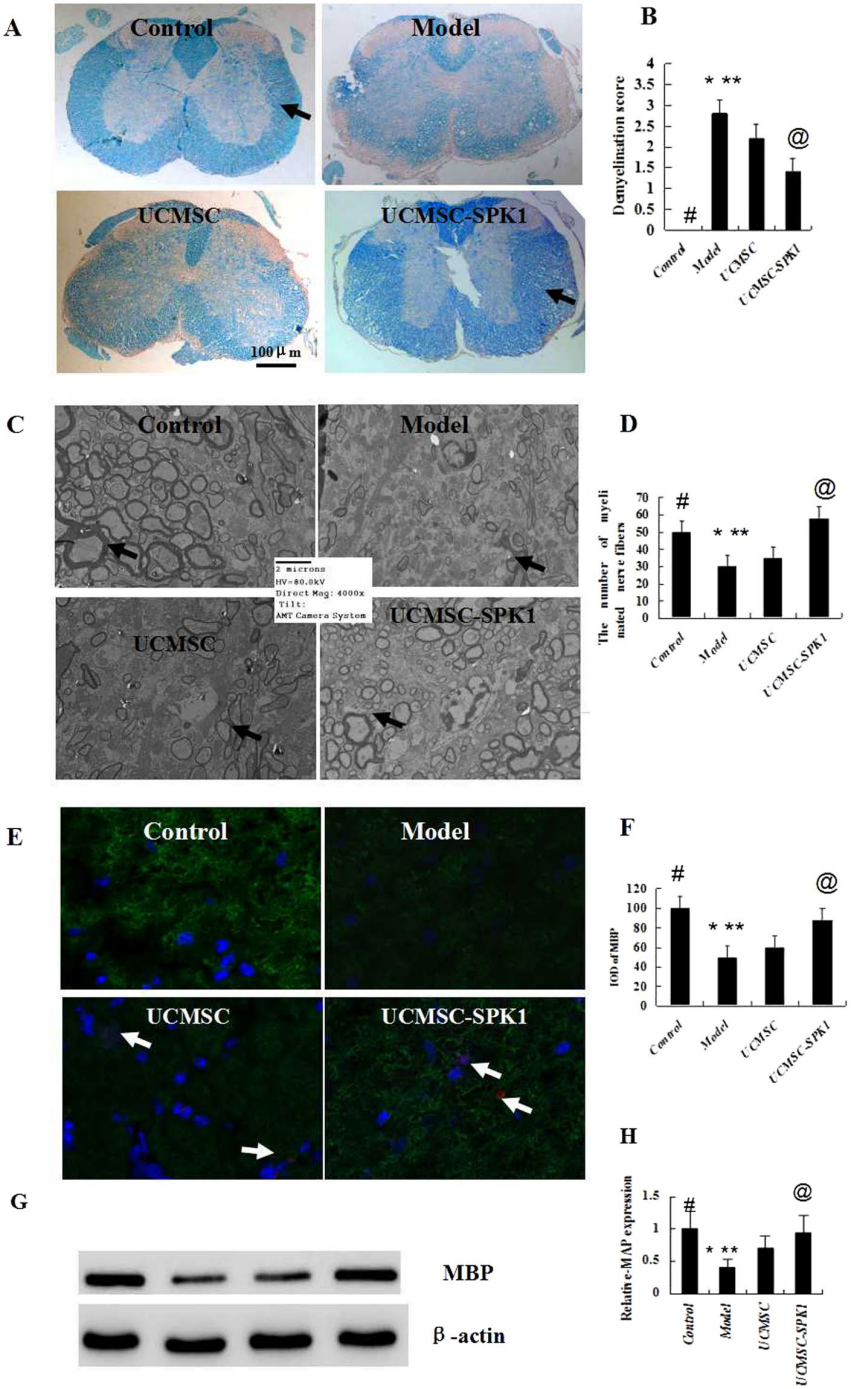
The observation of demyelination. (**A**) The white matter of the normal mice was dark blue, and the model group presented light blue. Demyelination was obviously improved in the UCMSC-SPK1 mice group as compared to that of UCMSC mice group. While compared to the normal mice, the myelinated fibers in UCMSC-SPK1 group were more sharpe (as shown by the arrow). (**B**) The demyelination score (^*#*^*P* < 0.01 the control vs the model group, *P < 0.05 the model group vs UCMSC group, **P < 0.01 the model group vs UCMSC-SPK1 group, ^*@*^*P* < 0.05 UCMSC group vs UCMSC-SPK1 group). (**C**) There were a lot of myelinated fibers in the white matter of the normal mice, while sharply decreased in the model group (as shown by the arrow) under TEM. After UCMSC-SPK1 treatment, the myelinated fibers significantly increased. (**D**) The number of myelinated fibers in the ten fields were calculated and statistically analysed, the tendency was consistent with that of the demyelination score of LFB staining. (**E**,**F**) The determination of the Confocal, and the tendency was consistent with that of the demyelination score of LFB staining. And the MSCs labelled by red fluorescence were also observed in the spinal cords of the UCMSC and UCMSC-SPK1 groups (as shown by the arrow). (**G**,**H**) The results of WB also powerfully proved this phenomenon.

After LFB staining, we observed that white matter of the normal mice was dark blue, and the model group presented light blue. While the kind of phenomenon was obviously improved in the UCMSC-SPK1 group as compared to that of UCMSC group (Fig. 3A). Although it was obvious that the myelinated fibers in UCMSC-SPK1 group were basically same to that in the normal mice, but those were more sharpe (as shown by the arrow). The demyelination score was shown in Fig. 3B (^*#*^*P* < 0.01 the control vs the model group, *P < 0.05 the model group vs UCMSC group, **P < 0.01 the model group vs UCMSC-SPK1 group, ^*@*^*P* < 0.05 UCMSC group vs UCMSC-SPK1 group). It was more clear under TEM there were a lot of myelinated fibers in the white matter of the normal mice, while sharply decreased in the model group (as shown by the arrow). After UCMSC-SPK1 treatment, the myelinated fibers significantly increased (Fig. 3C). The number of myelinated fibers in the ten fields were calculated and statistically analysed (Fig. 3D), and the trend was same to results of the LFB staining. Furthermore, the determination of the Confocal (Fig. 3E,F) and Western blot (Fig. 3G,H) powerfully proved these phenomena. And the MSCs labelled by red fluorescence were also observed in the spinal cords of the UCMSC and UCMSC-SPK1 groups (Fig. 3E, as shown by the arrow).

### The expression of GFAP in the spinal cords

**Figure 4:**
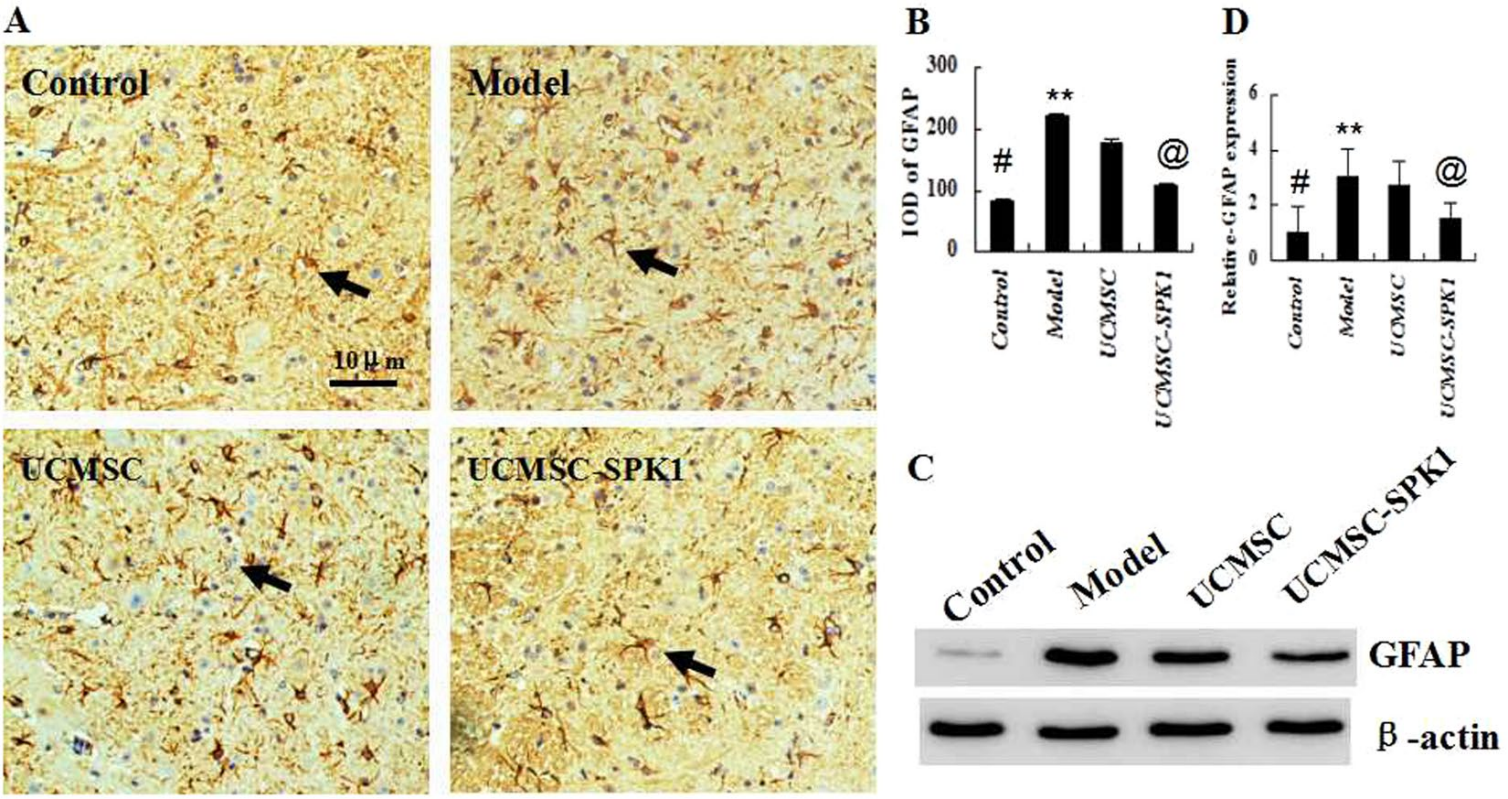
The expression of GFAP in the spinal cords. (**A**,**B**) Contrasting to the control, the level of GFAP in the model group significantly increased (^*#*^*P* << 0.01). After UCMSC-SPK1 treatment, the expression of GFAP obviously decreased (**P < 0.05), significantly difference was found in GFAP expression between the UCMSC-SPK1 and UCMSC group (^*@*^*P* < 0.05). (**C**,**D**) The western blot results indicated that GFAP expression tendency was consistent with that of the immunohistochemistry results.

To investigate the levels of GFAP expression in spinal cord of EAE mice, immunohistochemistry and western blot were performed. Contrasting to the control group, the level of GFAP in the model group significantly increased (^*#*^*P* < 0.01). After UCMSC-SPK1 treatment, the expression of GFAP obviously decreased (**P < 0.05), significantly difference was found in GFAP expression between the UCMSC-SPK1 and UCMSC group (^*@*^*P* < 0.05, Fig. 4A,B). The western blot results indicated that GFAP pression tendency was consistent with that of the immunohistochemistry results (Fig. 4C,D).

### FCM analysis of splenic NK and Treg cells

**Figure 5:**
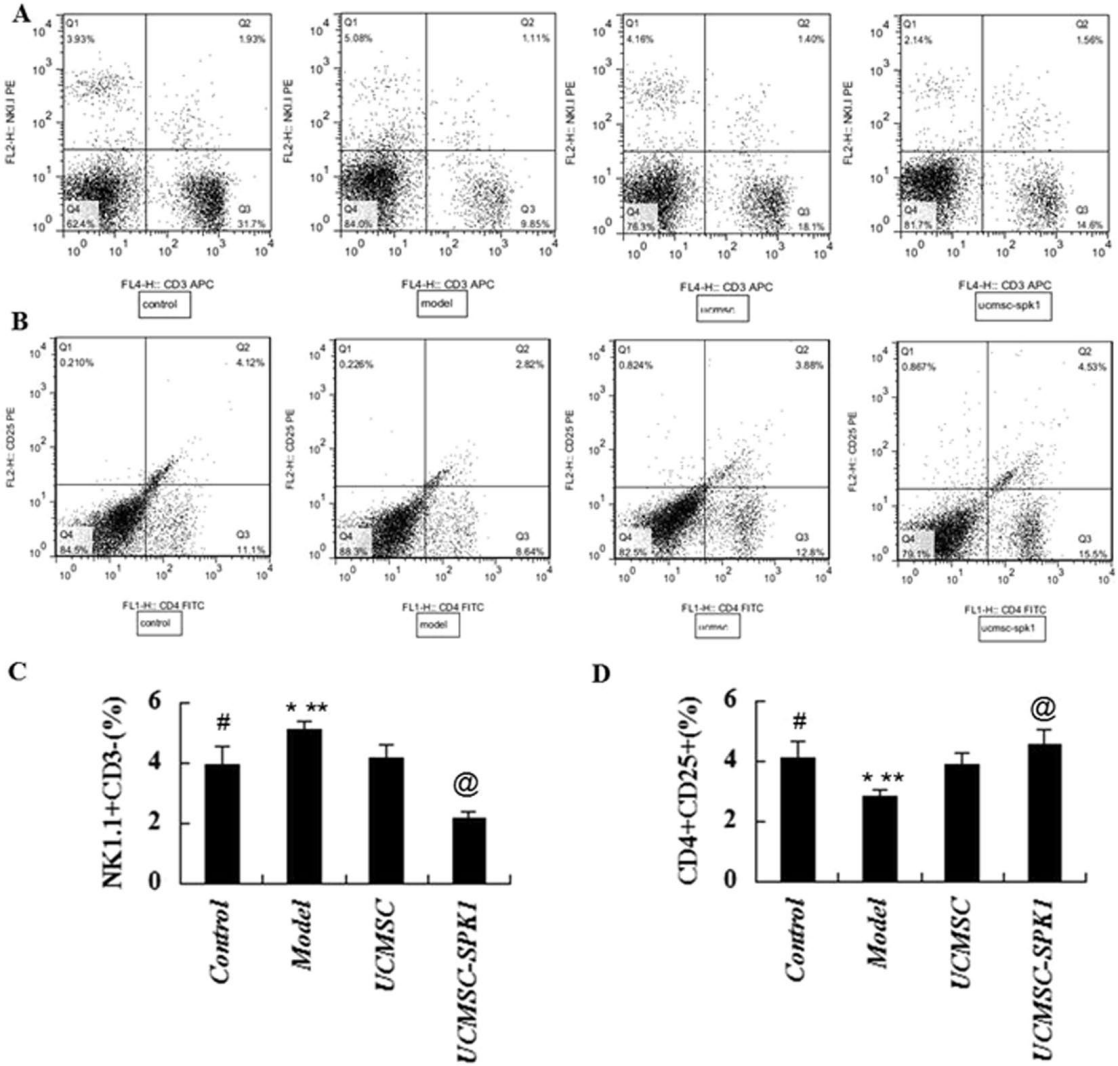
The levels of NK1.1CD3− and CD4+ CD25+ T cells were measured by FCM. The results showed that UCMSC and UCMSC-SPK1 treatment down-regulated markedly the ratio of NK cells, and increased the ratio of Treg cells in the spleen (*P < 0.05, **P < 0.01). According to the statistical analysis, the phenomena was more obvious in UCMSC-SPK1 group than that of UCMSC group (^*@*^*P* < 0.05) (^*#*^*P* < 0.05 the control group vs the model group).

To assess the changes in splenic NK and Treg cells in EAE mice, the levels of NK1.1CD3− and CD4+ CD25+ T cells were measured by FCM. The results showed that UCMSC and UCMSC-SPK1 treatment down-regulated markedly the ratio of NK cells, and increased the ratio of Treg cells in the spleen (*P < 0.05, **P < 0.01). According to the statistical analysis, the phenomena was more obvious in UCMSC-SPK1 group than that of UCMSC group (^*@*^*P* < 0.05, Fig. 5).

### ELISA analysis of inflammation-related cytokines in serum

**Figure 6:**
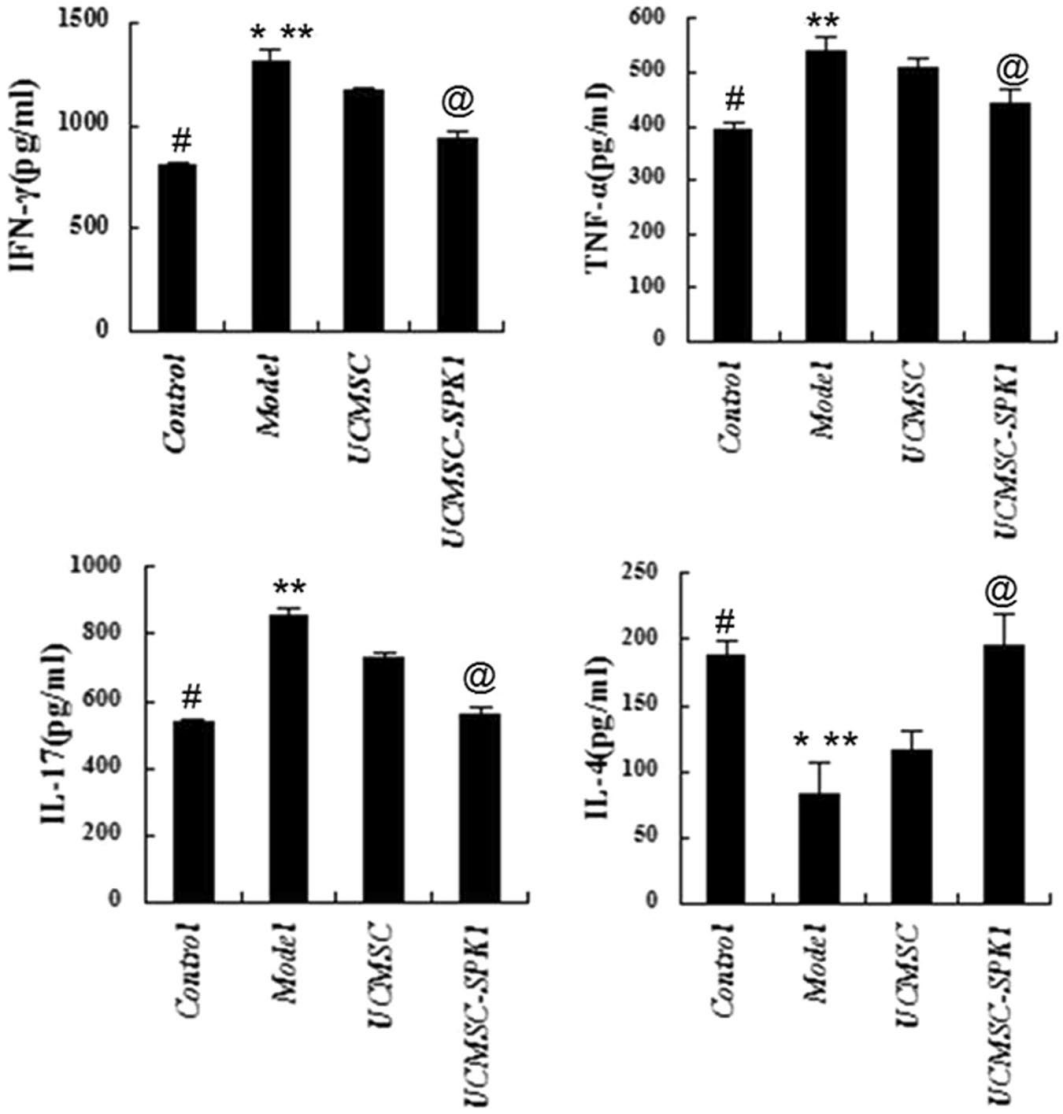
The levels of cytokines in EAE mice serum. Compared to control mice, the levels of IFN-γ, TNF-α, and IL-17 in the serum of EAE mice were significantly increased, while gradually declined after UCMSC and UCMSC-SPK1 treatment (*P < 0.05, **P < 0.05). There were significantly difference between UCMSC-SPK1 and UCMSC group (^*@*^*P* < 0.05). The level of IL-4 in EAE group was lower than that in UCMSC and UCMSC-SPK1 group (*P < 0.05, **P < 0.05), there was still significantly difference between the two groups (^*@*^*P* < 0.05) (^#^*P* < 0.05 the control group vs the model group).

Compared to control mice, the levels of IFN-γ, TNF-α, and IL-17 in the serum of EAE mice were significantly increased, while gradually declined after UCMSC and UCMSC-SPK1 treatment (*P < 0.05, **P < 0.05). It was obvious that the role of UCMSC-SPK1 was significantly stronger than that of UCMSC (^*@*^*P* < 0.05). The level of IL-4 in EAE group was lower than that in UCMSC and UCMSC-SPK1 group, there was still significantly difference between the two groups (^*@*^*P* < 0.05, Fig. 6).

## Discussion

Emerging data suggested that MSC transplantation represents an attractive therapeutic approach for MS treatment^9–11^. The human umbilical cord-derived MSCs have clear therapeutic potential, by suppressing the autoimmune response in early phases of disease in EAE patients. In our past study, no significant adverse effects were found in MS patients treated by UCMSC transplantation, accompanied by lower relapse occurrence and EDSS scores during a one-year observation period, in part, giving more evidence for the potential of UCMSC as a therapy for MS^22^.

However, appropriate gene modification may make MSC having more biological activity. In Natalie’s report, adipose-derived MSCs transduced with a bicistronic lentiviral vector encoding mouse IL-4 genetically engineered to express anti-inflammatory cytokines, facilitating immunomodulation and tissue protection in MS^23^. Gao and his team transplanted atglial cell line-derived neurotrophic factor (GDNF)-modified NSCs (GDNF/NSCs) and native NSCs into each lateral ventricle of rats. The results suggest that GDNF enhanced therapeutic efficiency of NSCs-based therapy for EAE^24^.

In this study, UCMSC was transfected by SPK1. The biological function of SPK1 mainly depends on its production of S1P, a sphingolipid metabolite that playing important roles in angiogenesis, inflammation, and cell growth, also, a key mediator in immune cell trafficking^18–20^. That is to say, SPK1/S1P signaling pathway has been implicated in many pathological processes, such as MS and cancer.

As is well known, FTY720 is an orally active S1P receptor modulator with a structure closely related to sphingosine and is shown to be highly effective in various autoimmune disease models including MS^20,25–27^. In EAE animal models, therapeutic or prophylactic administration of FTY720 reduces the infiltration of lymphocytes into the spinal cord with a rapid reduction in lymphocyte numbers in the peripheral blood produced by sequestration of lymphocytes within primary and secondary lymphoid organs^25–27^. However, FTY720 is oral drug, the misoperation of gavage usually results in the death of EAE mice. So we put FTY720 into the drinking water of mice every day, actually does not guarantee the dose of FTY720. And the effect of FTY720 is relatively poor than that of UCMSC-SPK1, even UCMSC in preliminary experiments (Data not shown). Therefore, the purpose of this study was to assess whether SPK1 would enhance therapeutic effect of UCMSC for EAE, SPK1 gene-modified UCMSC and native UCMSC were transplanted into the EAE mice through the tail vein.

In EAE and MS, autoreactive T cells migrate from peripheral tissues into the CNS where they are reactivated, thereby triggering an inflammatory cascade that results in extensive loss of myelin and myelinating cells (oligodendrocytes) as well as damage to axons and neurons^1–3^. In the present study, UCMSC transfected by SPK1 could significantly improve neurological functional recovery in EAE mice, than that in UCMSC group. According to the results of HE and LFB, reductions in EAE clinical scores were paralleled by reductions in demyelination, axonal loss, and astrogliosis in UCMSC-SPK1 group. It is most important that UCMSCs labelled by DiI were observed under confocal microscopy, proveing that UCMSCs could enter inside the brain of mice and play a powerful role.

In the adult CNS, astrocytes is the major cell type influencing EAE-associated behavioral, histological, and biochemical endpoints. This astrocyte identification is consistent with astrogliosis, identified by GFAP immuno labeling^28,29^. In EAE group, prominent astrogliosis was identified by increased GFAP immunoreactivity along with increased spinal cord cell density. Remarkably, UCMSC-SPK1 treatment prevented or reduced astrogliosis accompanying increases in cell density as did specific deletion of GFAP.

To analyze the neuroprotective mechanisms of UCMSC-SPK1, we investigated the development of NK and Treg responses in the spleen of EAE mice. It is worth noting that the ratio of Treg cells was obviously increased, and the ratio of NK cells decreased in UCMSC-SPK1 group than that of in UCMSC group. Treg cells play a crucial role in the maintenance of peripheral immune tolerance and can regulate effecter T cell-mediated autoimmune inflammation directly or via the antigen-presenting cells during MS/EAE^30,31^. In the present study, the increased population of Treg cells in the spinal cord from EAE mice was further increased by UCMSC-SPK1 treatment than UCMSC treatment. The canonical cytokine pattern in EAE is generated predominantly from Th1 and Th17 proinflammatory cells^31^. In our study, transplantation of UCMSC-SPK1 suppressed NK cells proliferation in recall cultures stimulated with MOG_35–55_ and decreased the production of pro-inflammatory cytokines, including IFN-γ, TNF-α and IL-17. The pro-inflammatory cytokines attack the myelin and axon, consequently, demyelination and axonal injury occur and result in the onset of MS/EAE.

In summary, we have shown that transplantation of UCMSCs gene modified by SPK1 dramatically reduce the severity of neurological deficits of the EAE mice, and can inhibit MOG-specific T-cells, which are at the fulcrum of EAE progression in C57Bl/6 mice immunized with MOG_35–55_. Furthermore, we describe that a shift in the cytokine response from Th1/Th17 to Th2 is an underlying mechanism that suppressed CNS autoimmunity. Therefore, UCMSCs overexpressing SPK1 gene potentially offer a novel mode for the treatment of MS. And the specific mechanism of SPK1 in treating MS/EAE will be an interesting research enterprise.

## Materials and Methods

### Reagents and antibodies

Adenovirus expressing green fluorescent protein gene (Ad-GFP) was provided by Beckman Medical Instruments, U.S.A. Recombinant adenovirus carrying SPK1 gene was constructed in our lab (Ad-SPK1). Umbilical cord tissue was obtained from the Department of Gynecology, the Second Affiliated Hospital of Zhengzhou University. All donors have signed informed consent and the study was approved by the ethics committee of the Second Affiliated Hospital of Zhengzhou University. Animal breeding, care and all experiments were performed in adherence to the animal experiment center guidelines published by the US National Institutes of Health, and approved by Animal Ethics Committee of the 148 Central Hospital of PLA, Beijing Institute of Radiation Medicine, and the Chinese PLA General Hospital. Confirming that all experiments were performed in accordance with relevant guidelines and regulations.

All chemicals and reagents, such as incomplete Freund’s adjuvant (IFA), pertussis toxin (PT) and polyclonal antibodies to glial fibrillary acidic protein (GFAP) and myelin basic protein (MBP) were purchased from Sigma Chemical Co. (Saint Louis, MO, USA). Myelin oligodendrocyte glycoprotein _35–55_ amino acid peptide (MOG_35–55,_ MEVGWYRSPFSRVVHLYRNGK) was synthesized by Bio-Scientific Co., Ltd (Shanghai, China), with a purity greater than 95%. Mycobacterium tuberculosis H37RA was purchased from Difco (Detroit, MI, USA). Polyclonal antibodies to SPK1 and GFAP, was purchased from Abcam (MA, USA). The anti-mouse natural killer (NK)-1.1, cluster of differentiation 3 (CD3), CD4, CD25 was purchased from Biolegend Inc (San Digeo, USA), enzyme linked immunosorbent assay (ELISA) Detection Kit was purchased from eBiosciences (San Diego, CA).

### HUC-MSC-SPK1 preparation

Isolation and verification of UCMSC were carried out based on previously described protocols, and determination on Ad-SPK1 optimal transfection efficiency was performed based on protocols described previously^13,14,32^. The UCMSCs were infected by Ad-SPK1 at 200 MOI for 48 hours, and then the cells were harvested. Concentrations of immunoreactive SPK1 in the supernatant were measured by ELISA. The SPK1 protein was quantified by BCA assay. After being electrophoresed on 10% sodium dodecyl sulfate polyacrylamide gel electropheresis (SDS-PAGE) gel, the proteins were transferred onto FluoroTrans^®^ W polyvinylidene fluoride (PVDF) membranes (Pall, 20685) via electrophoretic transfer system (Bio-Rad). Then the membranes were blocked with 5% skim milk in PBST for 1 hr followed by incubation with respective primary antibodies at 4 °C overnight. After thoroughly washed with PBST, the membranes were further incubated with respective horseradish peroxidase conjugated secondary antibodies. Thereafter, the protein bands were visualized with ECL-prime kit.

### EAE induction and treatment groups

Female mice (C57BL/6 J; 16–20 g, 6–8 weeks) were obtained from PLA Military Academy of Medical Science (Beijing, China), and allowed 7 days to acclimate before the start of the study.

EAE was induced as described previously^33,34^. Briefly, C57BL/6 mice were anesthetized with ketamine and xylazine and were immunized in the flanks with MOG_35–55_-CFA (2 mg/1 ml), which was made by emulsifying MOG_35–55_ in IFA supplemented with 5 mg/ml mycobacterium tuberculosis H37Ra on day 0 and day 7 in incomplete Freund’s adjuvant. Additionally, 200 ng of Pertussis toxin (Sigma, St Louis, MO) was given on day 0 and day 1 by i.p. injection. EAE clinical symptom was monitored daily and was graded according to the following common scale 0–5: 0, no clinical signs; limp tail or waddling gait with tail tonicity, 1; waddling gait with limp tail (ataxia), 2; ataxia with partial limb paralysis, 2.5; full paralysis of one limb, 3; full paralysis of one limb with partial paralysis of second limb, 3.5; full paralysis of two limbs, 4; moribund stage, 4.5; and death, 5.

48 mice were randomly assigned to four treatment groups as follows: (1) control group: equal volume of normal saline (NS) substituted for MOG_35–55_-CFA and PT to immunize mice and followed by saline treatment; (2) model group: EAE was induced by MOG_35–55_-CFA as described above and follow by saline treatment; (3) UCMSC group: after the EAE immunization, UCMSC (5.5 × 10^6^) were injected via the lateral tail vein in a volume of 100 µl on the day 7, 14, 21, 28; (4) UCMSC-SPK1 group: UCMSC-SPK1 (5.5 × 10^6^) were injected according to the same method as above.

### UCMSC and UCMSC-SPK1 were labelled by the carbocyanine dyes DiI

In order to detect whether the MSCs could enter into the spinal cord of the mice, the cells were labelled by the carbocyanine dyes DiI in the last lethal injection. Suspend cells at a density of 1 × 10^6^/mL in any chosen serum-free culture medium. Add 5 μL of the cell-labeling solution supplied per mL of cell suspension. Mix well by gentle pipetting. Incubate for 20 minutes at 37 °C. Centrifuge the labeled suspension tubes at 1500 rpm for 5 minutes, preferably at 37 °C. Remove the supernatant and gently resuspend the cells in warm (37 °C) medium. Repeat the wash procedure two more times. After treatment, the MSCs were labelled by DiI (red fluorescence) were detected by confocal microscopy (TissueFAXS plus, Austria), and MBP (dilution 1:100, Sigma, USA, green fluorescence) was observed at the same time.

### Histopathological analysis of EAE spinal cords

Six mice from every group were randomly assigned for the evaluation of histological analysis. Mice were killed on 33 days post-immunization (d.p.i.), the brain tissues and spinal cords were dissected out. The isolated brain tissues were immediately frozen in liquid nitrogen, and then stored at −80 °C for further analysis. The spinal cords were fixated in 4% formaldehyde, embedded in paraffin wax and sectioned. The sections were stained with Hematoxylin and Eosin (HE) to evaluate inflammatory cell infiltration. Histopathological examination was performed in a blinded fashion. The scale^35^ used to evaluate for inflammation was: 0, no inflammatory cells; 1, a few scattered inflammatory cells; 2, organisation of inflammatory infiltrates around blood vessels; and 3, extensive perivascular cuffing with extension into adjacent parenchyma or parenchymal infiltration without obvious cuffing.

### Luxol fast blue (LFB) staining

Demyelination of the spinal cord was assessed by LFB staining and scored as described^33,34^, with 0 indicating normal, 1 indicating one small focal area of demyelination, 2 indicating two or three areas, 3 indicating one to two large areas of demyelination, and 4 indicating extensive demyelination involving ≥20% of the white matter.

### Transmission electron microscope (TEM)

The spinal cord of the mice was fixed in formaldehyde at 4 °C for approximately 3 days. After dehydration, embedding and staining were performed, ultra structural and demyelination changes were observed under the TEM (HITACHI H-7650, Japan).

### Immunohistochemistry and analysis

Tissue sections were fixed in acetone and endogenous peroxidase activity was blocked by peroxidase block (0.3% hydrogen peroxide) for 15 min. Subsequently, the slides were incubated for 60 min at room temperature with one of the following primary antibodies: a rabbit polyclonal antibody directed against GFAP (dilution 1:500, Abcam, Cambridge, USA), a specific marker of astrocytes. Sections were incubated with appropriate peroxidase-linked secondary antibody. Staining, which was developed using a liquid 3,3′-diaminobenzidine (DAB) substrate kit, resulted in brown-colored precipitate at the antigen site. In negative controls, slides were incubated with PBS, in the absence of primary antibody. Finally, slides were counterstained with hematoxylin and mounted with Kaiser gel (Merck). The investigator who analyzed the slides was blinded to the fact from which experimental group the sections were obtained.

### Western blot (WB)

The spinal cord of 5 mice was dissected on ice and placed in lysis buffer; total tissue protein was extracted by repetitive frozen-thaw and centrifugation. Quantitative analysis of protein was made by using BCA-200 protein quantitative kit. 20 μg protein taken from each sample was mixed with loading buffer and dithiothreitol (DTT) at a ratio of 8:10:2, protein was denatured by boiling for 5 min, 12% SDS-PAGE gel was used for electrophoresis and membrane transfer. Nitrocellulose membrane was blocked with 5% skim milk for 1 h, thenincubated with rabbit anti-GFAP nd MBP antibody. After fully washing off uncombined antibody, the membrane was incubated with HRP-anti-rabbit antibody for 1 h; enhanced chemifluorescent staining was conducted. Quantity One software was applied for imaging analysis.

### Flow cytometry analysis

On day 33 p.i., mice were anesthetized, spleen and draining lymph nodes removed, and single cell suspension was prepared. Cells isolated from and spleens were adjusted and stained with PE anti-mouse NK-1.1, APC anti-mouse CD3; FITC anti-mouse CD4, PE anti-mouse CD25, for 30 min at 4 °C, then washed with PBS containing 1% FCS. All antibodies were obtained from BioLegend. In each test, 1 × 10^6^ cells were collected by a Canto II flow cytomerer using Cell Quest Diva software (BD Biosciences) and analyzed by FlowJo software (TriStar).

### Cytokine quantification

The serum of mice were harvested on 33 d.p.i. After being centrifuged at 4000 rpm for 10 min at 4 °C, the supernatants were subjected to further ELISA analysis. The concentrations of interferon-gamma (IFN-γ), tumor necrosis factor-alpha (TNF-α), interleukin-17 (IL-17), and interleukin-4 (IL-4) in brain homogenates were determined using respective ELISA kits referred to the manuals of manufacturer (eBiosciences, San Diego, CA). The cytokine concentrations of respective samples were quantified by standard curves prepared by recombinant cytokines of known concentrations.

### Statistical analysis

Statistical analyses were performed using SPSS10.0 software. Data presented as means ± SEM, were subjected to one- or two-way ANOVA, followed by either Newman-Keuls or Bonferroni’s multiple-comparisons test (as a post hoc test). *P* < 0.05 was considered to indicate statistical significance. The results of immunocytochemistry, confocal and western blot were analyzed by Image-Pro Plus 5.0 image analyzer (Media Cybernetics, USA). The integrated optical density (IOD) and gray values were assayed by statistical analysis.
